# Association between albumin corrected anion gap and 30-day all-cause mortality of critically ill patients with acute myocardial infarction: a retrospective analysis based on the MIMIC-IV database

**DOI:** 10.1186/s12872-023-03200-3

**Published:** 2023-04-28

**Authors:** Linhao Jian, Zhixiang Zhang, Quan Zhou, Xiangjie Duan, Haiqin Xu, Liangqing Ge

**Affiliations:** 1 Department of the First Clinical College, Jinan University, 601 Huangpu Avenue West, Guangzhou, 510632 China; 2 Department of Cardiology, The First People’s Hospital of Changde, 818 Renmin Avenue, Changde City, 415003 China; 3 Department of Science and Education, The First People’s Hospital of Changde, 818 Renmin Avenue, Changde City, 415003 China; 4 Department of Infectious Diseases, The First People’s Hospital of Changde, 818 Renmin Avenue, Changde City, 415003 China; 5 Department of Cardiac Electrophysiology, The First People’s Hospital of Changde, 818 Renmin Avenue, Changde City, 415003 China

**Keywords:** ACAG, 30 d all-cause mortality, MIMIC IV, Acute myocardial infarction, Cardiovascular disorder

## Abstract

**Background:**

The anion gap (AG) has been linked to the prognosis of many cardiovascular disorders. However, the correlation between albumin-corrected anion gap (ACAG) and 30 d all-cause mortality of intensive care patients with acute myocardial infarction (AMI) is unclear. Furthermore, owing to the lack of studies, it is also unknown whether ACAG is more accurate than AG in predicting the mortality of AMI.

**Methods:**

The Medical Information Mart for Intensive Care IV (MIMIC IV) dataset was used to provide patient data in this retrospective cohort study. ACAG is computed using the formulae: [4.4—{albumin (g/dl)}] × 2.5 + AG. The primary outcome was 30 d all-cause mortality intensive care patients with AMI. To explore the prognostic worthiness of ACAG, the receiver operating characteristic curve, smooth curve fitting, Cox regression model, and Kaplan survival analysis was performed.

**Results:**

We enrolled 2,160 patients in this study. ACAG had a better predictive value for 30 d all-cause mortality than AG, with an area under the curve of 0.66. The association between ACAG levels and overall mortality was nonlinear. In our model, after correcting for confounding factors, the ACAG was the independent predictor for 30 d all-cause mortality (HR 1.75, 95%CI 1.24, 2.47). ACAG K-M estimator curve analyses revealed that the group with ACAG ≥ 21.75 mmol/l had poor survival rate than the other group.

**Conclusions:**

High serum ACAG levels were a significant risk factor for 30 d all-cause mortality in critically ill patients with AMI. ACAG concentration and 30 d all-cause mortality had a nonlinear relationship. ACAG had better predictive value in identifying 30 d all-cause mortality of patients with AMI in ICU than the AG.

**Supplementary Information:**

The online version contains supplementary material available at 10.1186/s12872-023-03200-3.

## Background

Acute myocardial infarction (AMI) is still the leading cause of cardiac death globally. Mortality of patients with AMI is decreasing in most high-income countries as treatment levels improve [1, 2]. However, with the rising prevalence of obesity, diabetes, hypertension, and hyperlipidaemia, an increase in the incidence of AMI has been recognized globally, and the overall prognosis remains poor. The enormous burden of death caused by the high morbidity of AMI imposes significant challenges worldwide [3, 4]. Therefore, simpler and less expensive biomarkers to predict prognosis is preferred in patients with AMI admitted to the ICU.

The anion gap (AG) is a mathematical variable derived from the difference in serum cation and anion levels and is one of the most commonly used traditional markers. Metabolic acidosis is a common cause of an elevated AG level; typically, an elevated AG level is caused by excessive organic acid synthesis, including lactate and acetoacetate [5]. Blood lactate levels are elevated in most patients with AMI 2 h after symptoms appear [6]. Previous research has recommended serum AG in adult patients in ICU as a precise and sensitive predictor of prognosis or mortality [7]. AG is associated with increased mortality rates and acute kidney injury in patients in CCU, and it is an independent risk factor for in-hospital all-cause mortality [8]. AG has been linked to poor clinical outcomes and a high mortality rate in patients with coronary heart disease [9]. In critically ill patients with AMI, it was an independent risk factor for short-term all-cause mortality [10]. Sahu et al. [11] revealed that the presence of AG acidosis (AG ≥ 12 mmol/L) on admission was a strong predictor of short-term mortality in patients with AMI with and without cardiogenic shock. In another study of the long-term prognosis of AMI [12], higher AG levels were significantly associated with increased 1-year all-cause mortality compared to those with a normal AG. It may be useful for predicting cardiovascular mortality and risk stratification in patients with AMI as a readily available marker.

No albumin correction for AG was performed when analysing the value of AG for predicting the short-term all-cause mortality of critically ill patients with cardiac diseases, which may have resulted in bias in the results [10]. One of the major proteins in the human blood circulation system, serum albumin, is known as a conventional indicator of nutritional and inflammatory status. Serum albumin content has been shown to be an excellent predictor of adverse outcomes in patients with AMI [13, 14]. Hypoproteinemia may be associated with metabolic alkalosis, and hypoproteinemia may underestimate acidosis severity [15]. Hatherill et al. [16] maintained that albumin-corrected anion gap (ACAG), which is calculated using the formulae: [4.4—{albumin (g/dl)}] × 2.5 + AG, is the best diagnostic and screening tool for metabolic acidosis in the ICU. Tianyang Hu et al. [17] found that ACAG outperformed albumin and AG in predicting in-hospital mortality in ICU patients with sepsis.

However, whether the ACAG has a better predictive prognosis capability than AG in patients with AMI admitted to ICU is unknown. We speculated that complex markers would have an advantage over single markers. This study aimed to clarify the correlation between ACAG levels and 30 d all-cause mortality in patients with AMI in the ICU using real-world data and investigate whether ACAG is better than AG in predicting mortality.

## Methods

### Study population

We included adult patients with AMI based on the International Classification of Diseases, 9th revision (ICD-9) code of 410 or the International Classification of Diseases, 10th revision (ICD-10) code of I210.0-I214.0 or I219.0 (the ICD code of AMI) adopted in Medical Information Mart for Intensive Care IV (MIMIC IV) database. The following were the inclusion criteria: 1. patients admitted to the ICU with a primary diagnosis of AMI; 2. First admission to the ICU in the data resource; 3. Age ≥ 18 years; and 4. Stay in the ICU for ≥ 24 h. The exclusion criteria included following: 1. Lack of important data, such as anion gap, albumin level, 30 d mortality, and treatment-related information, among others. 2. Patients were admitted to the ICU multiple times; however, we only considered the initial ICU admission for each patient.

### Data source and extraction

The data for this study was obtained from the Medical Information Mart for Intensive Care IV (MIMIC IV) database. MIMIC-IV (edition 2.0) is an updated version of the MIMIC-III [18], comprising de-identified health-related information from individuals who lived in Beth Israel Deaconess Medical Centre critical care units between 2008 and 2019. After completing the web-based course at the National Institute of Health and passing the Protecting Human Research Participants examination, one of the authors obtained access to this information and was responsible for data extraction (certification number: 42039823). The Massachusetts Institute of Technology and BIDMC Institutional Review Board have approved the database. All patient data were anonymized to be exempted from informed consent. The study was designed in accordance with the Helsinki Declaration for studies involving humans. The study was approved by the medical ethics committee of our institution (Batch number: 2022–197-01).

The parameters retrieved include age, sex, ethnicity, comorbidities, laboratory variables, treatments, vital signs, weight, clinical outcome, and so on. Laboratory parameters include anion gap, albumin, creatinine, glucose, sodium, potassium, white blood cell (WBC), red blood cell (RBC), haemoglobin (Hb), platelet, and troponin-T, among others. All lab results were collected from data recorded within the first 24 h following the admission of the patients to the ICU.

### Definition of AMI

The term AMI should be used when there is an acute myocardial injury with clinical evidence of acute myocardial ischaemia and with the detection of a rise or fall of cTn values with at least one value above the 99th percentile URL and at least one of the following [19]:

- Symptoms of myocardial ischaemia.
- New ischaemic electrocardiogram changes.
- Pathological Q waves development.
- Imaging evidence of new loss of viable myocardium or new regional wall motion abnormality in a pattern consistent with an ischaemic aetiology.
- Identification of a coronary thrombus using angiography or autopsy.

### Definition of ACAG and the primary outcome

AG was calculated using the equation: AG (mmol/l) = (Na + K)—(Cl + HCO_3_), whereas ACAG was calculated using the Figge-Jabor-Kazda-Fencl equation: ACAG (mmol/l) = [4.4-observed albumin (g/dl)] * 2.5 + observed AG [16]. The primary endpoint of this study was 30 d post-AMI all-cause death.

### Statistical analysis

The median and interquartile range were used for continuous data, while frequency and percentage were used for categorical data. The chi-square analysis was used for categorical variables to compare groups, whereas the Kruskal–Wallis analysis was used for continuous variables. All individuals were categorized into three groups based on their ACAG tertiles distribution: 1st tertile (T1 < 17.50 mmol/l), 2nd tertile (T2 17.50–21.75 mmol/l), and 3rd tertile (T3 >  = 21.75 mmol/l). To assess the diagnostic ability of ACAG and AG, we used Delong et al. [20]. method to compare the area under the curve (AUC) of ACAG and AG.

For univariate and multivariate analyses, Cox regression was used to determine the relationship between the relevant influential variables and 30 d death. In the model I, factors were only adapted for age, sex, and ethnicity. Model 2 adapted for all parameters in model 1 and the medically relevant variables, including vital signs and laboratory results. The outcomes were expressed as a hazard ratio (HR) with a 95% confidence interval (CI). Nonlinear correlations were determined using a generalized additive model (GAM). The threshold effect of ACAG on 30 d mortality is calculated using a two-piecewise linear regression model according to the smoothing plot when a nonlinear correlation is observed. Kaplan–Meier (K-M) survival curves and the Log-rank test were used to describe survival distribution. We used stratified analysis to reveal whether the impact of ACAG differed between subgroups. R (v3.42) and Empower Stats v2.17.8 (http://www.empowerstats.com/cn/) were used to analyse all data. All stated *P*-values were two-sided, and two-tailed probability values of < 5% were considered statistically significant.

## Results

### Characteristics of patients

Overall, 2,160 patients with AMI were enrolled in this study, with 1,730 surviving and 430 dying within 30 d post-AMI. Patients were divided into three groups. The basic features of the three groups are based on their ACAG tertiles. Table 1 shows the basic characteristics of the three groups. T1, T2, and T3 each had 712, 706, and 742 patients in ICU, respectively. The median age was 72.60 years old (19.77–97.47), with female patients accounting for 41.25% (891/2160). The patients with ACAG ≥ 21.75 mmol/L were younger, had a higher requirement of mechanical ventilation and use of vasoactive agents, and had a greater rate of bleeding and 30 d all-cause mortality. Additional File Table 1 shows additional laboratory indicators.

**Table 1:** Individuals’ basic characteristics categorized based on ACAG

Characteristic	Total cohort (*n* = 2160)	Tertile of the ACAG			*P*
		< 17.50(712)	17.50–21.75(706)	> = 21.75(742)	
Age, years	72.60 (19.77–97.47)	72.80 (22.84–97.47)	73.25 (19.77–96.75)	71.53 (21.65–97.42)	0.011
Female, n (%)	891 (41.25%)	277 (38.90%)	302 (42.78%)	312 (42.05%)	0.288
Ethnicity, n %)					< 0.001
White	1413 (65.42%)	468 (65.73%)	484 (68.56%)	461 (62.13%)	
Black	205 (9.49%)	51 (7.16%)	61 (8.64%)	93 (12.53%)	
Asian	46 (2.13%)	9 (1.26%)	14 (1.98%)	23 (3.10%)	
Unknown	496 (22.96%)	184 (25.84%)	147 (20.82%)	165 (22.24%)	
AF, n(%)	821 (38.01%)	251 (35.25%)	287 (40.65%)	283 (38.14%)	0.111
Hypertension, n(%)	753 (34.86%)	300 (42.13%)	258 (36.54%)	195 (26.28%)	< 0.001
Hyperlipidemia, n(%)	1102 (51.02%)	397 (55.76%)	356 (50.42%)	349 (47.04%)	0.004
CCI	7.00 (1.00–20.00)	7.00 (1.00–18.00)	8.00 (1.00–20.00)	8.00 (1.00–17.00)	< 0.001
MV, n(%)	919 (42.55%)	276 (38.76%)	270 (38.24%)	373 (50.27%)	< 0.001
Vasoactive drugs, n(%)	1129 (52.27%)	312 (43.82%)	359 (50.85%)	458 (61.73%)	< 0.001
β-blockers, n(%)	1136 (52.59%)	427 (59.97%)	362 (51.27%)	347 (46.77%)	< 0.001
CABG, n(%)	294 (13.61%)	173 (24.30%)	82 (11.61%)	39 (5.26%)	< 0.001
PCI, n(%)	357 (16.53%)	141 (19.80%)	126 (17.85%)	90 (12.13%)	< 0.001
Heart rate, bpm	88.00 (34.00–179.00)	82.00 (40.00–157.00)	89.00 (34.00–179.00)	93.00 (41.00–179.00)	< 0.001
SBP, mmHg	119.00 (46.00–229.00)	121.00 (65.00–204.00)	119.00 (47.00–229.00)	118.00 (46.00–220.00)	0.023
DBP, mmHg	67.00 (11.00–190.00)	68.00 (18.00–171.00)	66.00 (11.00–176.00)	66.00 (11.00–190.00)	0.290
Weight, Kg	78.70 (33.60–231.50)	78.12 (35.70–201.00)	79.00 (35.80–231.50)	78.10 (33.60–181.70)	0.756
SPO_2_, %	97.00 (14.00–100.00)	98.00 (14.00–100.00)	97.00 (62.00–100.00)	97.50 (59.00–100.00)	0.041
Albumin, g/dl	3.40 (0.80–5.40)	3.50 (1.20–5.00)	3.30 (1.00–5.20)	3.20 (0.80–5.40)	< 0.001
AG, mmol/L	17.00 (5.00–56.00)	13.00 (5.00–18.00)	17.00 (11.00–22.00)	22.00 (15.00–56.00)	< 0.001
CK-MB, U/L	11.00 (1.00–673.00)	12.00 (1.00–594.00)	10.00 (1.00–575.00)	12.00 (1.00–673.00)	0.196
Troponin-T, ng/ml	0.44 (0.01–51.84)	0.43 (0.01–24.98)	0.44 (0.01–41.30)	0.47 (0.01–51.84)	0.350
Outcome, n(%)					
Stroke	36 (1.67%)	10 (1.40%)	12 (1.70%)	14 (1.89%)	0.770
Bleeding	298 (13.80%)	77 (10.81%)	93 (13.17%)	128 (17.25%)	0.002
30-day mortality	430 (19.91%)	81 (11.38%)	125 (17.71%)	224 (30.19%)	< 0.001

### ROC curve analysis

**Fig. 1:**
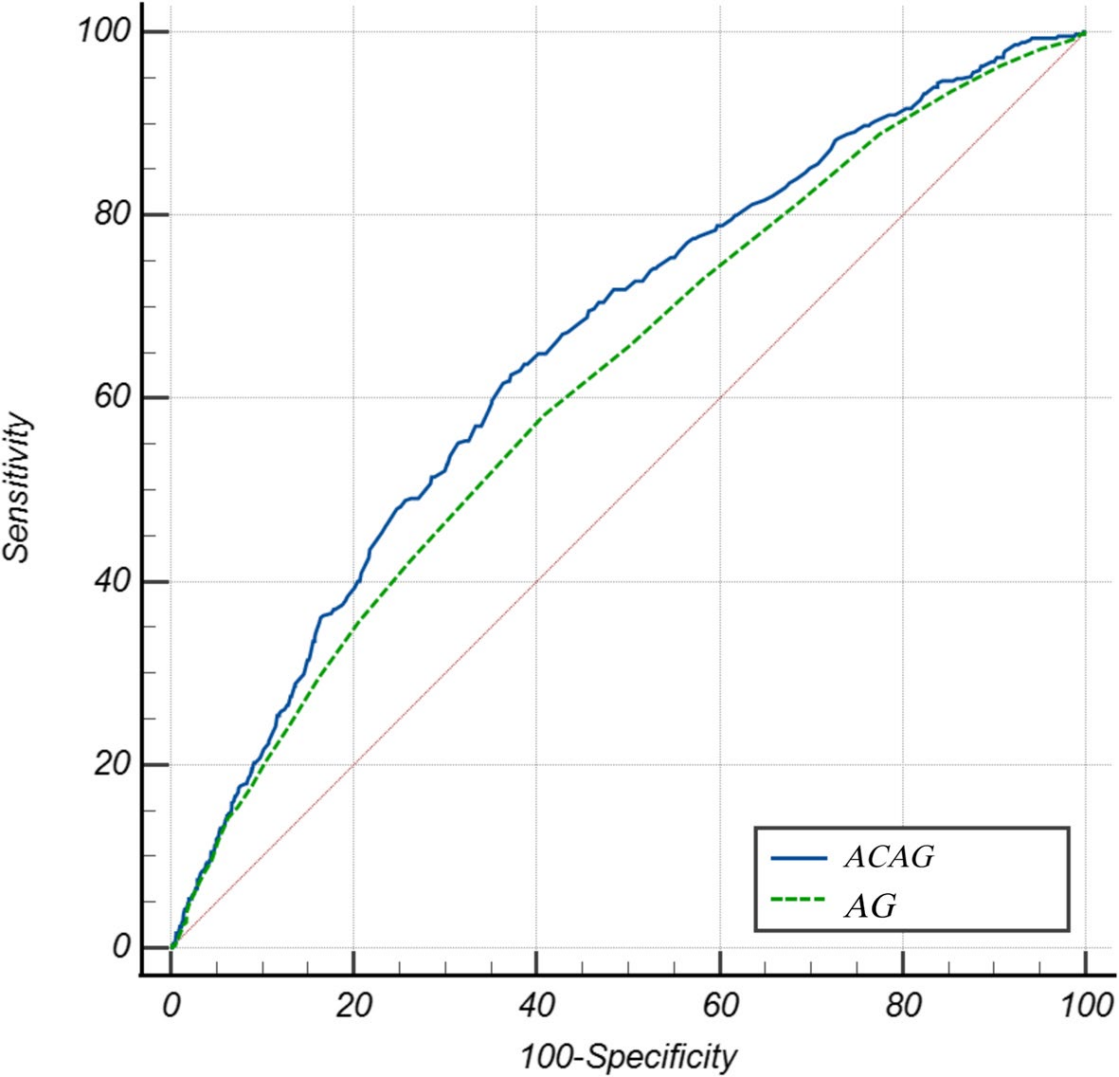
Receiver-operating characteristic curve of the ACAG and AG to predict 30 -day all-cause mortality of AMI

The ACAG prediction capability was evaluated using receiver operating characteristic (ROC) curve analysis. The AUCs (95% CI) for ACAG and AG were 0.66 (0.63, 0.69) and 0.62 (0.59, 0.65), respectively (Fig. 1). ACAG and AG had sensitivity, specificity, and best thresholds of 0.63, 0.63, 20.5, 0.58, 0.59, and 17.5, respectively. In addition, we observed that ACAG predicted 30 d all-cause mortality better than AG (Z = 6.785, *p* < 0.01).

### Univariate and multivariate analyses

Table 2 shows the results of univariate and multivariate Cox regression analyses. Age, atrial fibrillation, Charlson Comorbidity Index, WBC, use of mechanical ventilation, Troponin-T, vasoactive drug use, and ACAG were found to be positively correlated with all-cause mortality in univariate analyses. However, hyperlipidaemia, systemic blood pressure (SBP), diastolic blood pressure (DBP), SPO_2_, β-blockers use, CABG surgery, and PCI were all negatively correlated with mortality. The multivariate Cox regression included statistically significant variables screened from the univariate analysis. Age, troponin-T, mechanical ventilation use, vasoactive drugs use, and ACAG were still positively correlated with mortality in the multivariate Cox regression model. Only hyperlipidaemia, β-blockers use, CABG, and PCI were negatively associated with mortality.

**Table 2:** Univariate and Multivariate analyses of factors correlated with 30-day all-cause mortality

Variable	Univariate analysis		Multivariate analysis	
	HR (95% CI)	*P*	HR (95% CI)	*P*
Age	1.02 (1.01, 1.03)	< 0.0001	1.03 (1.02, 1.04)	< 0.0001
Heart rate	1.00 (1.00, 1.01)	0.2612	NA	
SBP	0.99 (0.99, 1.00)	0.0005	1.00 (0.99, 1.01)	0.9085
DBP	0.99 (0.99, 1.00)	0.0401	1.00 (0.99, 1.00)	0.3891
SPO_2_	0.98 (0.96, 0.99)	0.0015	0.99 (0.97, 1.01)	0.3898
CCI	1.06 (1.02, 1.09)	0.0016	1.01 (0.97, 1.06)	0.5739
Creatinine	1.00 (0.97, 1.04)	0.9957	NA	
Glucose	1.00 (1.00, 1.00)	0.4938	NA	
Sodium	1.01 (1.00, 1.03)	0.1645	NA	
Potassium	1.08 (0.99, 1.18)	0.0723	NA	
WBC	1.01 (1.00, 1.01)	0.0038	1.01 (1.00, 1.01)	0.1439
Platelets	1.00 (1.00, 1.00)	0.0063	1.00 (1.00, 1.00)	0.1125
RBC	0.94 (0.84, 1.05)	0.2976	NA	
ALT	1.00 (1.00, 1.00)	0.0331	1.00 (1.00, 1.00)	0.9075
AST	1.00 (1.00, 1.00)	0.3072	NA	
CK-MB	1.00 (1.00, 1.00)	0.1253	NA	
Troponin-T	1.04 (1.02, 1.07)	0.0003	1.05 (1.02, 1.07)	0.0001
ACAG	1.05 (1.04, 1.07)	< 0.0001	1.03 (1.01, 1.05)	0.0009
Female	1.00 (0.83, 1.22)	0.9683	NA	
AF	1.32 (1.09, 1.59)	0.0044	1.14 (0.92, 1.43)	0.2359
Hypertension	0.90 (0.73, 1.10)	0.3084	NA	
Hyperlipidemia	0.68 (0.56, 0.82)	< 0.0001	0.78 (0.63, 0.97)	0.0258
Strock	1.07 (0.59, 1.96)	0.8153	NA	
Bleeding	1.03 (0.80, 1.32)	0.8350	NA	
Vasoactive drugs	2.88 (2.29, 3.62)	< 0.0001	2.09 (1.57, 2.78)	< 0.0001
β-blockers	0.59 (0.48, 0.71)	< 0.0001	0.69 (0.56, 0.86)	0.0008
CABG	0.26 (0.16, 0.42)	< 0.0001	0.28 (0.16, 0.51)	< 0.0001
PCI	0.69 (0.50, 0.95)	0.0211	0.58 (0.39, 0.85)	0.0059
MV	2.04 (1.68, 2.49)	< 0.0001	1.41 (1.11, 1.79)	0.0049

*Abbreviations: HR* Hazard ratio, *CI* Confidence interval

### Subgroup assessment of the correlation between the ACAG and 30-day all-cause death

A Sub-group assessment was used to explore the relationship of ACAG with the 30 d all-cause death rate (Table 3). Further, no significant interaction was observed in most strata (*p* = 0.10–0.54).

**Table 3:** The associations of ACAG with 30-day all-cause mortality in stratified subgroups

Variables	n	Hazard ratio (95%CI)	*P* for interaction
Age			0.0016
< 65	648	1.03 (1.00, 1.06)	
> = 65	1512	1.08 (1.06, 1.10)	
Gender			0.2110
male	1269	1.06 (1.04, 1.08)	
female	891	1.04 (1.02, 1.06)	
Ethnicity			0.3356
White	1413	1.06 (1.04, 1.08)	
Black	205	1.02 (0.99, 1.06)	
Asian	46	1.07 (1.00, 1.15)	
Unknown	496	1.06 (1.04, 1.09)	
MV			0.5439
no	1241	1.04 (1.01, 1.06)	
yes	919	1.05 (1.03, 1.07)	
Vasoactive drugs			0.4838
no	1031	1.03 (0.99, 1.06)	
yes	1129	1.05 (1.03, 1.06)	
β-blockers			0.1030
no	1024	1.06 (1.04, 1.08)	
yes	1136	1.03 (1.00, 1.05)	
CABG			0.0007
no	1866	1.04 (1.03, 1.06)	
yes	294	1.20 (1.11, 1.29)	
PCI			0.1275
no	1803	1.05 (1.03, 1.06)	
yes	357	1.09 (1.04, 1.15)	
Hypertension			0.1872
no	1407	1.06 (1.04, 1.08)	
yes	753	1.04 (1.01, 1.06)	
Bleeding			0.4720
no	1862	1.05 (1.04, 1.07)	
yes	298	1.04 (1.01, 1.08)	
Albumin			0.5480
> = 3.5	967	1.04(1.02, 1.07)	
< 3.5	1193	1.05(1.04, 1.07)	

*Abbreviations: HR* Hazard ratio, *CI* Confidence interval

### Relationship of ACAG with outcome

The multivariate cox regression analysis revealed that higher ACAG was related to 30 d all-cause death (Table 4). After adjusting for the clinical confounders, ACAG levels (per 1 mmol/l increase) were linked to a 5% increased risk of 30 d all-cause mortality rate (HR 1.05, 95% CI, 1.02, 1.07, *p* < 0.0001). In the adjusted II model, patients in the T3 group (ACAG ≥ 21.75 mmol/l) had an increased risk of 30 d all-cause mortality (HR 1.75, 95%CI 1.24, 2.47, *p* < 0.0013) compared to the T1 group (ACAG 17.50 mmol/l). However, there was no significant variation in the T2 between the T1 (*P* > 0.05). It is easy to see that the correlation between ACAG and 30 d all-cause death was nonlinear.

**Table 4:** Association between different ACAG levels and 30-day all-causes mortality with AMI

	ACAG (mmol/l)		
	HR (95% CI)	*P* value	*P* for trend
Unadjusted			< 0.0001
T1: ACAG < 17.50	Ref		
T2: 17.50 ≤ AG < 21.75	1.35 (1.02, 1.79)	0.0339	
T3: ACAG ≥ 21.75	2.22 (1.72, 2.87)	< 0.0001	
Continuous	1.05 (1.04, 1.07)	< 0.0001	
Modle I			< 0.0001
T1: ACAG < 17.50	Ref		
T2: 17.50 ≤ AG < 21.75	1.36 (1.03, 1.80)	0.0307	
T3: ACAG ≥ 21.75	2.38 (1.84, 3.07)	< 0.0001	
Continuous	1.07 (1.05, 1.08)	< 0.0001	
Modle II			< 0.0005
T1: ACAG < 17.50	Ref		
T2: 17.50 ≤ AG < 21.75	1.17 (0.83, 1.66)	0.3640	
T3: ACAG ≥ 21.75	1.75 (1.24, 2.47)	0.0013	
Continuous	1.05 (1.02, 1.07)	< 0.0001	

Unadjusted model adjust for: None

Modle I adjust for: age, gender, ethnicity

Modle II adjust for: age, gender, ethnicity, weight, AF, hyperlipidemia, CCI, MV, heart rate, SBP, DBP, SPO_2_, Creatinine, Glucose, Sodium, Potassium, Platelets, WBC, RBC, Hb, ALT, CK-MB, Hypertension, Stroke, Bleeding, Vasoactive drugs, β-blockers, Troponin-T, CABG, PCI

*Abbreviations: HR* Hazard ratio, *CI* Confidence interval

**Fig. 2:**
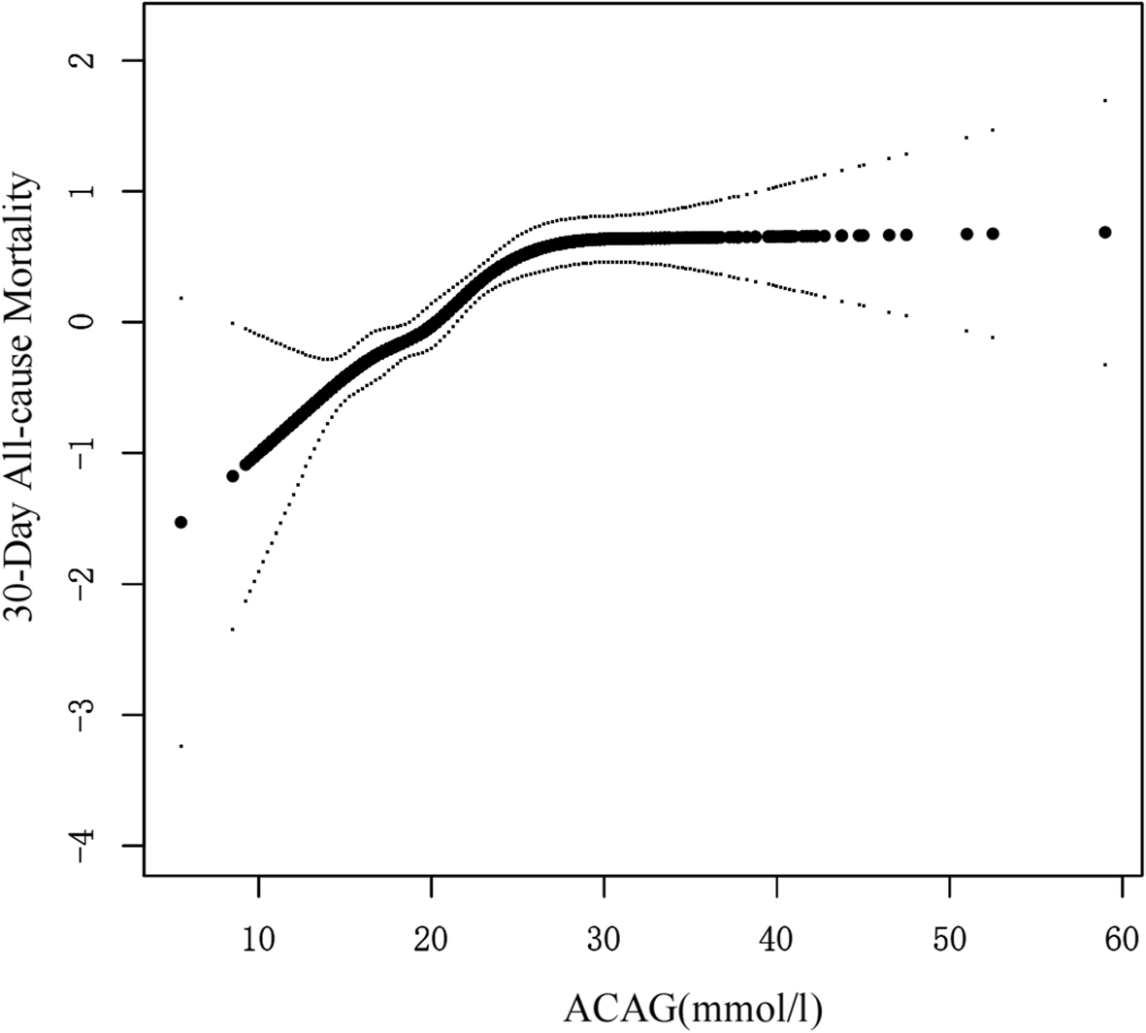
The smoothing curves illustrating the association between ACAG and 30-day all-cause mortality

After adjusting for possible confounding factors, smooth curve fitting was performed. We confirmed that the correlation between ACAG concentration and 30 d all-cause death was nonlinear (Fig. 2) after adjusting for age, sex, ethnicity, weight, atrial fibrillation, hyperlipidaemia, Charlson Comorbidity Index, mechanical ventilation requirement, heart rate, SBP, DBP, SPO_2_, creatinine, glucose, sodium, potassium, platelets, WBC, RBC, Hb, ALT, CK-MB, hypertension, stroke, bleeding, vasoactive drugs use, β-blockers use, Troponin-T, CABG, and PCI. We calculated the inflection point as 24.5 using two-piecewise linear regression and a recursive algorithm (Table 5). ACAG was positively correlated with 30 d all-cause death to the left of the inflection point. There was no increased mortality on the right of the inflection point as ACAG levels increased.

**Table 5:** Threshold effect analysis of ACAG on 30-day all-cause mortality in ICU individuals with AMI using the two-piecewise model of linear regression

30-day all-cause mortality	Adjusted HR	95%CI	*P* value
Fitting by the standard linear model	1.05	(1.04, 1.07)	< 0.0001
Fitting by the two-piecewise linear model			
Inflection point	24.5		
ACAG < 24.5(mmol/l)	1.11	(1.07, 1.14)	< 0.0001
ACAG ≥ 24.5(mmol/l)	1.01	(0.99, 1.04)	0.3520
Log likelihood ratio			< 0.001

Adjust for: age, gender, ethnicity, weight, AF, hyperlipidemia, CCI, MV, heart rate, SBP, DBP, SPO2, Creatinine, Glucose, Sodium, Potassium, Platelets, WBC, RBC, Hb, ALT, CK-MB, Hypertension, Stroke, Bleeding, Vasoactive drugs, β-blockers, Troponin-T, CABG, PCI

*Abbreviations: HR* Hazard ratio, *CI* Confidence interval

**Fig. 3:**
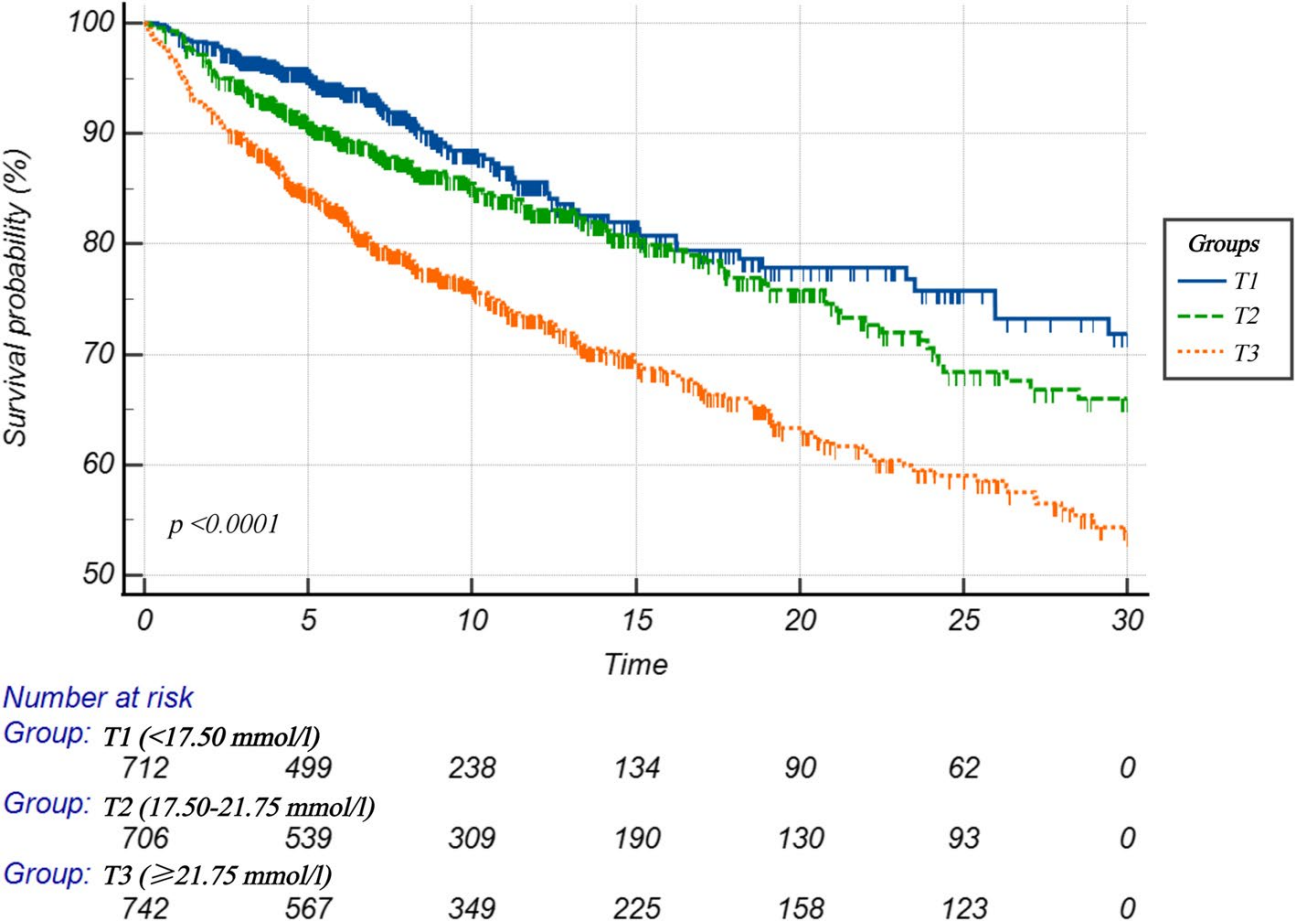
Kaplan–Meier curves of the ACAG for evaluating 30-day all-cause mortality of AMI

The Kaplan–Meier analysis stratified by ACAG tertiles reveals a link between ACAG levels and 30 d mortality (Fig. 3). In addition, we found that patients with the highest levels of ACAG (≥ 21.75 mmol/l) had significantly lower survival than those with low levels (*P* < 0.0001).

## Discussion

In this retrospective study, we found a nonlinear correlation between ACAG concentration and 30 d all-cause death in critically ill patients with AMI. Higher ACAG was linked to a higher risk of 1-month all-cause death. In contrast, at ACAG levels around ≥ 24.5 mmol/L, the risk of 1-month all-cause death no longer increased. Furthermore, the ROC curve analysis revealed that the ACAG had a higher predictive value than the AG in identifying 1-month all-cause death of patients with AMI in the ICU.

A prospective cohort study revealed that patients with coronary artery disease (CAD) who had AG ≥ 15.92 mmol/L had a 5.17-fold higher risk of all-cause mortality after 30 d, and higher AG was related to worse cardiac function [9]. In a study of 171 patients with AMI, AG acidosis presence was correlated with an increased mortality risk (OR 4.2, 95% CI 2.3–7.5) [11]. According to Tienan Sun et al., AG was an independent risk factor for in-hospital all-cause death and was related to worse medical outcomes in CCU patients with CAD [8]. In a separate study of critically ill patients with AMI, the correlation between AG and all-cause death was linear for 6- and 12 months and nearly linear for 30 d death, with higher levels correlated with increased all-cause death [12].

However, albumin has an effect on AG levels, and the excellent value of AG necessitates albumin correction [21, 22]. Owing to the fact, that albumin makes up a large fraction of the unmeasured anions, any change in albumin levels resulted in alterations in AG levels. Serum albumin levels usually drop sharply in critically ill patients admitted to the ICU, and albumin levels increase with disease remission [23]. Hypoproteinemia was found in approximately 55% of patients with AMI in the ICU in our study. Durward et al. [24] found that approximately half of the high anion gap metabolic acidosis would be missed without correction for albumin. According to research [25], each 1 g/L albumin level reduction reduces the AG concentration by 2.3–2.5 mmol/L. However, AG is suggested to be implicated in many cardiovascular diseases and it seems to worsen the prognosis of patients with cardiovascular diseases [9, 11, 26]. Therefore, albumin correction may be required to investigate the relationship between AG and the prognosis of AMI, especially in patients with hypoproteinemia.

Hence, it is critical to investigate the role of ACAG in critically ill patients with AMI and the relationship between ACAG and AMI prognosis. A study of patients with sepsis revealed that ACAG had the most significant prognostic validity for in-hospital mortality of ICU patients with sepsis, outperforming albumin and AG [17]. Hagiwara et al. [27] published a study involving 166 patients with cardiopulmonary arrest. They found that ACAG outperforms AG in predicting the resumption of spontaneous circulation following cardiopulmonary resuscitation in patients with cardiopulmonary failure. Similarly, we found that the ACAG estimated 30 d mortality of patients with AMI in the ICU outperformed AG. We revealed a nonlinear association between ACAG concentrations and 30 d all-cause death, unlike the studies above. However, the precise mechanism is unknown. We speculate that the nonlinear relationship reflects the real scenario of the prognostic value of AG in critically ill patients with AMI. Critically ill patients with AMI frequently develop hypoproteinemia, putting them at risk for an acid–base imbalance. Therefore, using ACAG to evaluate the 30 d all-cause death of critically ill patients with AMI has a high predictive value.

We speculated that increased ACAG levels reduced the short-term mortality of AMI primarily through AG acidosis. Increased anion gap acidosis is typically caused by excessive organic acid production or the simultaneous and proportional decrease in anions and net acid elimination observed in various forms of kidney failure [28, 29]. However, the pathophysiologic basis for the development of AG acidosis in patients with AMI is not completely understood. We speculated that AG acidosis might be related to pump failure following AMI, tissue hypoperfusion, and RAAS activation. Owing to the fact that acidosis induces vasodilation, it reduces peripheral resistance and systolic blood pressure [30]. The adverse effects of acidosis on cardiac function have been well documented, with the reduced cardiac output characterized as the hemodynamic profile. Sahu et al. [11] believe that a compensatory respiratory alkalosis could influence pH; nonetheless, the AG is a reliable biomarker of metabolic dysregulation that is relatively independent of acute respiratory alterations. We analysed the ACAG rather than lactic acid levels or arterial pH to ensure the authenticity of the results and ease of implementation. To the best of our knowledge, this is the first study to assess the effect of ACAG on the short-term prognosis of patients admitted to the ICU following AMI. We suggest that clinicians pay attention to the effect of hypoproteinemia on AG and that using ACAG in prognostic judgment may be more accurate. Researchers should pay attention to ACAG when constructing prognostic models of AMI.

This study had some limitations. First, due to the retrospective status of the study, selection bias cannot be completely excluded. The proportion of patients with hypertension was low, while those with unclear racial information were high (Table 1). Second, because the study was observational, the cause-effect relationship of ACAG on AMI all-cause mortality was not clarified. Third, ACAG levels were only assessed once and not for their dynamic changes. Fourth, the study included patients with AMI admitted to the ICU. These findings may not apply to all patients with AMI and prevalent coronary heart disease. Fifth, even though we used a large number of covariates to control for confounding in the multivariate Cox proportional hazards model, other unstudied confounders could have influenced our findings. Finally, due to the limitations of the MIMIC-IV database, we were unable to obtain comprehensive hospitalization data of the patients, such as cardiac function classification, echocardiogram results, specific location of myocardial infarction, thrombolysis in myocardial infarction risk score, Global Registry of Acute Coronary Events risk score, and other data that may affect the prognosis of patients. Further studies are required to verify the findings of this study. Nevertheless, despite the limitations, our study is important in understanding the relationship between ACAG and AMI.

## Conclusions

The high serum ACAG levels were a significant risk factor for 30 d all-cause mortality in critically ill patients with AMI. ACAG concentration and 30 d all-cause mortality had a nonlinear relationship. ACAG had better predictive value in identifying 30 d all-cause mortality of patients with AMI in ICU than the AG. In summary, ACAG is inexpensive and easy to obtain, and it can potentially improve the initial risk stratification and early interventional therapeutic strategies of patients with AMI.

## Supplementary Information

**Additional file Table 1.** Baseline laboratory features categorized based on ACAG.

## Data Availability

The MIMIC IV database (version 2.0) is publically available from https://mimic-iv.mit.edu/. Any researcher who adheres to the data use requirements is permitted access to the database.

## Abbreviations

AG: Anion gap
CVD: Cardiovascular disorders
ACAG: Albumin-corrected anion gap
AMI: Acute myocardial infarction
ROC: Receiver operating characteristic
AUC: Area under the curve
MIMIC IV: Mart for Intensive Care IV
WBC: White blood cell
RBC: Red blood cell
Hb: Haemoglobin
HR: Hazard ratio
SBP: Systemic blood pressure
DBP: Diastolic blood pressure
CAD: Coronary artery disease
CCI: Charlson Comorbidity Index
MV: Mechanical ventilation
CABG: Coronary artery bypass grafting
PCI: Percutaneous coronary intervention
CK-MB: Creatine kinase-MB
ALT: Alanine transaminase
AST: Aspartate transaminase
AF: Atrial fibrillation
