# Supplementary material for: Association between albumin corrected anion gap and 30-day all-cause mortality of critically ill patients with acute myocardial infarction: a retrospective analysis based on the MIMIC-IV database
Source: BMC Cardiovasc Disord. 2023 Apr 28;23:211. doi: 10.1186/s12872-023-03200-3 (PMC10148465; doi:10.1186/s12872-023-03200-3)
Supplement: Supplementary file 1 — Additional file Table 1. Baseline laboratory features categorized based on ACAG. [file 12872_2023_3200_MOESM1_ESM.doc]

Supplementary Table 1 Baseline laboratory features categorized based on ACAG.

| Characteristic | Total cohort  (n=2160) | Tertile of the ACAG | | |  |
| --- | --- | --- | --- | --- | --- |
|  |  | <17.50(712) | 17.50-21.75(706) | >=21.75(742) | P |
| Creatinine, mg/dl | 1.40 (0.20-43.00) | 1.00 (0.30-6.80) | 1.40 (0.30-11.40) | 2.00 (0.20-43.00) | <0.001 |
| Glucose, mg/dl | 148.00 (20.00-1630.00) | 135.00 (39.00-1200.00) | 150.00 (20.00-805.00) | 173.00 (39.00-1630.00) | <0.001 |
| Sodium, mmol/L | 138.00 (102.00-185.00) | 138.00 (116.00-159.00) | 138.00 (102.00-185.00) | 137.00 (105.00-172.00) | <0.001 |
| Potassium, mmol/L | 4.40 (1.70-9.80) | 4.20 (1.90-9.80) | 4.30 (1.90-9.50) | 4.60 (1.70-9.60) | <0.001 |
| WBC, k/mcl | 12.00 (0.10-185.40) | 10.80 (0.10-185.40) | 11.80  (0.50-61.80) | 13.60 (0.10-123.80) | <0.001 |
| Platelets, k/mcl | 210.00 (11.00-1160.00) | 204.50 (13.00-774.00) | 210.00 (11.00-1160.00) | 216.00 (11.00-878.00) | 0.279 |
| RBC, m/mcl | 3.69 (0.68-6.84) | 3.92 (1.09-6.46) | 3.60 (1.03-6.08) | 3.54 (0.68-6.84) | <0.001 |
| Hb, g/dl | 11.00  (2.20-18.30) | 11.60  (3.10-17.50) | 10.80  (2.70-18.30) | 10.50 (2.20-17.60) | <0.001 |
| ALT, U/L | 31.00 (2.00-10569.00) | 28.00 (5.00-5166.00) | 31.00 (5.00-7240.00) | 38.50 (2.00-10569.00) | <0.001 |
| AST, U/L | 55.00 (2.00-27999.00) | 45.00 (7.00-7400.00) | 54.00 (2.00-11270.00) | 70.00 (6.00-27999.00) | <0.001 |
